# Phosphosite Mapping of *HIP-55* Protein in Mammalian Cells

**DOI:** 10.3390/ijms15034903

**Published:** 2014-03-19

**Authors:** Ning Liu, Ningning Sun, Xiang Gao, Zijian Li

**Affiliations:** 1 Institute of Vascular Medicine, Peking University Third Hospital, Key Laboratory of Cardiovascular Molecular Biology and Regulatory Peptides, Ministry of Health, Key Laboratory of Molecular Cardiovascular Sciences, Ministry of Education and Beijing Key Laboratory of Cardiovascular Receptors Research, Beijing 100191, China; 2 Central Laboratory, Jilin University Second Hospital, Changchun 130041, China; E-Mails: liu_ning@jlu.edu.cn (N.L.); sunnn13@mails.jlu.edu.cn (N.S.); gaoxiang13@mails.jlu.edu.cn (X.G.)

**Keywords:** mass spectrometry, *HIP-55*, phosphorylation

## Abstract

In the present study, hematopoietic progenitor kinase 1 (HPK1)-interacting protein of 55 kDa (*HIP-55*) protein was over-expressed in HEK293 cells, which was genetically attached with 6x His tag. The protein was purified by nickel-charged resin and was then subjected to tryptic digestion. The phosphorylated peptides within the *HIP-55* protein were enriched by TiO_2_ affinity chromatography, followed by mass spectrometry analysis. Fourteen phosphorylation sites along the primary structure of *HIP-55* protein were identified, most of which had not been previously reported. Our results indicate that bio-mass spectrometry coupled with manual interpretation can be used to successfully identify the phosphorylation modification in *HIP-55* protein in HEK293 cells.

## 1. Introduction

Protein phosphorylation is a fundamental type of post-translational modification, which plays a significant role in a wide range of cellular processes. Reversible phosphorylation results in a conformational change in the structure of many enzymes, receptors and adaptor proteins, triggering cellular signaling transduction, and modulating protein function, stability, interaction and localization. [1,2] Phosphorylation usually occurs on serine, threonine and tyrosine in eukaryotic proteins. [3] Phosphorylation on serine is the most common, followed by threonine and tyrosine. Determining protein phosphorylation sites is often the first step in the elucidation of a biological mechanism. Within a protein, phosphorylation can occur on several amino acids. The different phosphorylation sites mediate different biological processes. Therefore, the identification of *in vivo* phosphorylated sites of proteins is extremely important for understanding biological function and processes.

*HIP-55*, also called *SH3P7*, *mAbp1* and *DBNL*, is a multi-domain adaptor protein, with an actin-binding domain at its *N*-terminus and an SH3 domain at its *C*-terminus [4]. *HIP-55* acts as adaptor protein in many cellular processes such as cell signaling transduction and receptor endocytosis [5,6]. *HIP-55* also plays important roles in T-cell proliferation, immune responses and the development of cerebellar architecture [7–10]. Some important functions of the *HIP-55* protein are reported as mediated and regulated by its phosphorylation. *HIP-55* was identified as a tyrosine kinase substrate using anti-phosphotyrosine antibodies [11]. *HIP-55* is phosphorylated by Syk, Lyn, and Blk and further links antigen receptor signaling to components of the cytoskeleton [12]. *HIP-55* is also identified as a novel MELK substrate and is important for stem-cell characteristics and invasiveness [13]. Furthermore, src-mediated phosphorylation of *HIP-55* regulates podosome rosette formation in transformed fibroblasts [14]. Clearly, the *HIP-55* protein is involved in many signal transduction processes; thus, investigation of its phosphorylation modifications is important to better understand the role of *HIP-55* protein in the precise regulation of signal transduction.

Traditionally, three approaches are used to determine phosphorylation sites: the bioinformatics approach, the biochemical approach, and the genetics approach. In the last decade, advancements in mass spectrometry have redefined conventional biochemical approaches for the identification of phosphorylation sites. Mass spectrometry (MS) has become currently the most powerful technique for analysis of phosphorylation sites [15,16]. In the present study, mass spectrometry combined with phosphopeptide enrichment techniques were employed to identify the phosphorylation sites of *HIP-55* protein in mammalian cell. *HIP-55* protein carrying a tag of six histidines was over-expressed in HEK293 cells, which was enriched by Ni-NTA resin. Reverse capillary high efficiency liquid chromatography (HPLC) coupled with mass spectrometry was employed to profile phosphorylation modifications in the purified *HIP-55* protein. Several novel sites of phosphorylation in *HIP-55* protein were identified.

## 2. Results and Discussion

### 2.1. Expression and Purification of His-Tagged HIP-55 Protein

**Figure 1.**
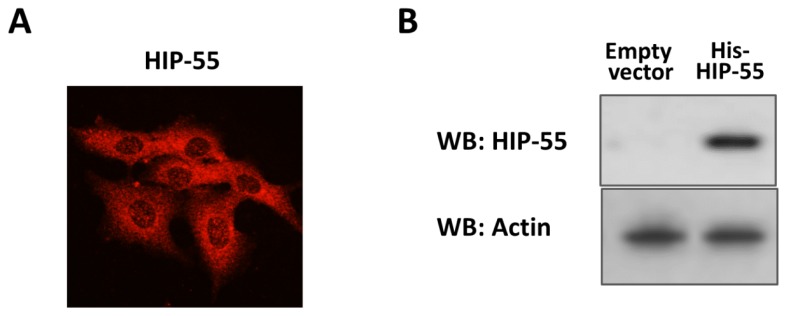
Expression and purification of *HIP-55* protein. (**A**) Cells were fixed and labeled with antibodies to *HIP-55*. Subcellular location of *HIP-55* was shown by laser scanning confocal microscopy; (**B**) After His-pull-down, the overexpression of *HIP-55* in the HEK293 cell line was detected with Western blot assay by *HIP-55* antibody. Actin was used as a loading control.

*HIP-55*, as an adaptor protein, was found to be expressed in various mammalian cells. The distribution of *HIP-55* was examined with an immunofluorescence assay. The result showed that *HIP-55* was observed throughout the cytosol and appeared enriched in the perinuclear area (Figure 1A). pDEST-His-*HIP-55* plasmid with full length *HIP-55* gene attached to a six-histidine tag were transfected into HEK293 cells which were maintained in DMEM containing 10% fetal bovine serum for 36 h at 37 °C. After the medium was removed, cells were washed with PBS three times. Then the cells were harvested and lyzed. *HIP-55* protein was purified with Ni-NTA resin and further precipitated with chilled acetone to remove high amounts of salts such as guanidine HCl and imidazole. The protein pellet was collected and dissolved in SDS-containing buffer. The purified *HIP-55* protein was checked by western blot analysis as indicated in Figure 1B.

### 2.2. Identification of HIP-55 Protein by MS

**Figure 2.**
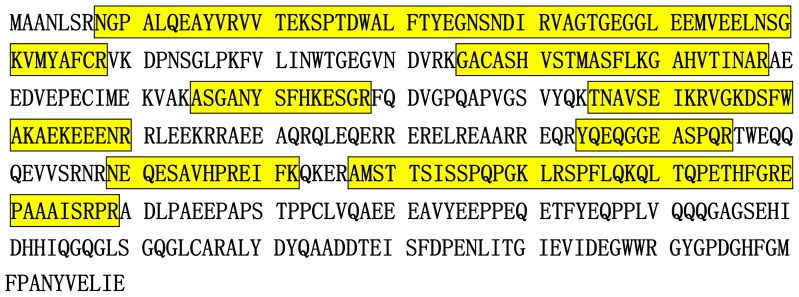
Primary structure of *HIP-55* protein. The tryptic peptides identified by LC-MS/MS analysis are highlighted.

The eluted proteins from Ni-NTA resin were desalted by acetone precipitation and then subjected to tryptic digestion. Prior to the analysis of phosphorylation, the peptide mixture was directly subjected to LC-MS/MS analysis, and the obtained data were inputted into a protein database for searching. Results showed a series of tryptic peptides from *HIP-55* protein with a coverage rate of 45.1%, in which only one phosphopeptide (LRS*PFLQK) was identified when Ser, Thr and Tyr phosphorylations were considered during the database searching. Figure 2 shows the distribution of peptides in the identification of *HIP-55* protein.

### 2.3. MS Analysis of Phosphorylation Sites of HIP-55 Protein

By enrichment of phosphopeptides from tryptic peptides mixture with TiO_2_, a total of fourteen phosphopeptides from *HIP-55* protein were identified by LC-MS/MS analysis and database searching, including the one previously detected without TiO_2_ enrichment (Table 1).

A few spectra were given below as examples for the identification of some of these phosphorylation sites in *HIP-55* protein.

**Figure 3.**
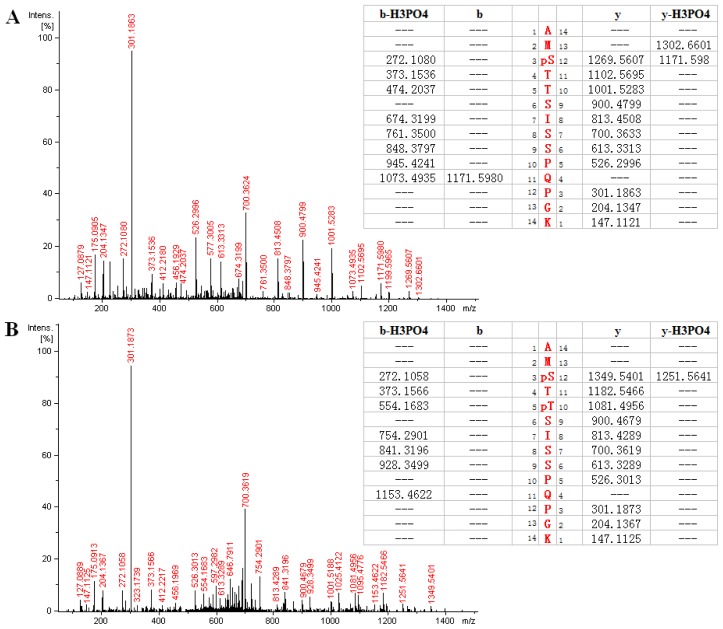
(**A**) MS/MS spectrum of a doubly-charged peak at *m*/*z* 736.333. The corresponding peptide is identified as AMpSTTSISSPQPGK (267–280), of which Serine 269 is phosphorylated; (**B**) MS/MS spectrum of a doubly-charged peak at *m*/*z* 776.318. The corresponding peptide is identified as AMpSTpTSISSPQPGK (267–280), of which both Serine 269 and Threonine 271 are phosphorylated.

Figure 3A showed the MS/MS spectrum of a doubly-charged molecular ion peak with *m*/*z* at 736.333. Database searching identified a phosphopeptide as AMpSTTSISSPQPGK (267–280) from *HIP-55* protein. A series of fragment ions such as fragment ions of *b* and *y* series can be clearly identified. The detection of *y* series ions at *m*/*z* 1102.5695, 1269.5607, 1171.5980 and 1302.6601 indicated that phosphorylation occurred exclusively at Serine 269 in this peptide. Detection of *b* series ions also confirmed this identification. Figure 3B shows the spectrum of a doubly-charged molecular ion peak with *m*/*z* at 776.318, which is similar to Figure 3A. The corresponding peptide is identified as the same peptide sequence as in Figure 3A, but with an additional phosphorylation site at Threonine 271. As shown in Figure 3B, *y* series ions at *m*/*z* 900.4679, 1081.4956, 1182.5466 and 1349.5401 indicated that phosphorylation occurred exclusively at Serine 269 in this peptide. Detection of b series ions also confirmed this identification. As shown in both mass spectra, the base peak at *m*/*z* 301.19 was observed, which resulted from fragmentation at Proline 278. It was noticed that most of the b series ions were subject to neutral loss of 98 (a phosphate molecule).

**Figure 4.**
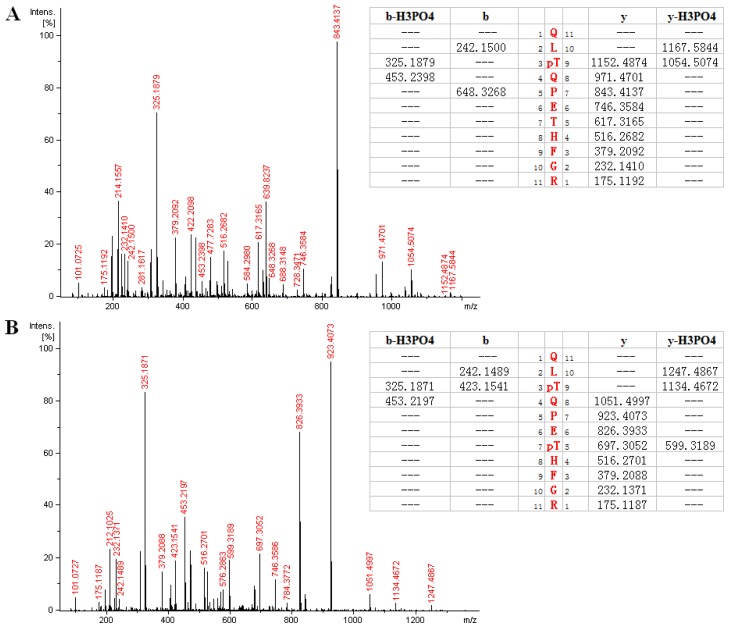
(**A**) MS/MS spectrum of a doubly-charged peak at *m*/*z* 697.321. The corresponding peptide is identified as QLpTQPETHFGR (289–299), in which Threonine 291 is phosphorylated; (**B**) MS/MS spectrum of a doubly-charged peak at *m*/*z* 737.304. The corresponding peptide is identified as QLpTQPEpTHFGR (289–299), in which both Threonine 291 and Threonine 295 are phosphorylated.

As another example, Figure 4A showed the MS/MS spectrum of a doubly-charged molecular ion peak with *m*/*z* at 697.321, from which a phosphopeptide as QLpTQPETHFGR(289–299) from *HIP-55* protein was identified by database searching. The assignment of *y* series ions at *m*/*z* 971.4701, 1054.5074, 1152.4874 and 1167.5844 clearly indicated phosphorylation at Threonine 291, which was confirmed by detection of b series ions at *m*/*z* 242.1500, 325.1871, 423.1541 and 453.2197. The base peak at *m*/*z* 843.4137 was from the fragmentation at Proline 293. Figure 4B shows a similar spectrum of a doubly-charged molecular ion peak with *m*/*z* at 737.304, which was identified as the same peptide sequence (289–299) with two phosphorylation sites at Threonine 291 and 295. The detection of *y* series ions at *m*/*z* 697.3052, 826.3933, as well as the base peak ion at *m*/*z* 923.4073, confirmed the identification.

**Figure 5.**
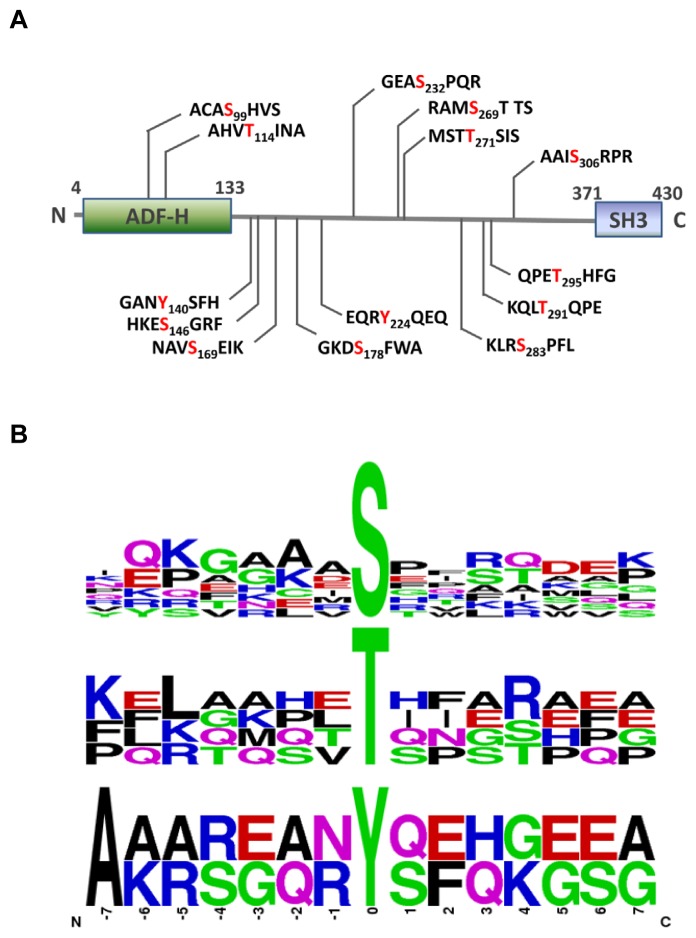
Graphical display and motif analysis of Phosphosites of *HIP-55* Protein. (**A**) Graphical display of phosphosite mapping of *HIP-55* Protein; (**B**) Frequency distribution of amino acid residues surrounding phosphorylation sites at positions −7 to +7.

The phosphosite mapping of *HIP-55* protein were graphically displayed as Figure 5A. Furthermore, motif analyses of phosphosites were created and displayed using the Weblogo server (http://weblogo.berkeley.edu/). A modification site consists of the modified residue at the 0 position, plus the seven flanking amino acids *N*-terminal (positions −7 to −1) and *C*-terminal (positions +1 to +7) to the modification site. For the analysis of sequence features adjacent to the identified phosphosites of *HIP-55* protein, the 14 amino acids surrounding each phosphosite were extracted and aligned. The motif logo shows the difference in the frequency of amino acids surrounding the different phosphosites (serine, threonine, and tyrosine), suggesting the kinase recognition motif of these three kinds of phosphorylation sites are different in mammalian cells (Figure 5B). In addition, our results showed that serine and threonine residues undergo phosphorylation more often than tyrosine residues. The phospho-amino acid content ratio (pSer:pThr:pTyr) was 9:4:2 within *HIP-55* protein, which was consistent with previous reports of that in the whole-cell [17,18].

Protein phosphorylation plays a significant role in a wide range of cellular processes. It is estimated that approximately one-third of all proteins in eukaryotic cells are phosphorylated at any given time [17]. Therefore, the mapping of phosphorylation sites of special interest proteins is the subject of a large body of research. Because of the prominent role of protein phosphorylation in signaling transduction, traditional studies focus on enzymes, receptors and ion channels which are switched “on” or “off” by phosphorylation and dephosphorylation in signaling transduction process [19–22]. However, one of the major goals of studying signal transduction is to determine the mechanisms that control cross-talk between signaling cascades and to determine how specificity in signaling is achieved. An emerging class of proteins that are major contributors to these processes are adaptor proteins [23]. By linking specific binding proteins together, adaptor proteins control cellular signals appropriately. Most adaptor proteins binding to partners function as signaling regulators in a phosphorylation-dependent manner. Therefore, identification of phosphorylation sites of adaptor proteins is of critical importance in the field of cellular signal transduction. *HIP-55*, as a signaling adaptor protein, has been shown as a common effector of antigen receptor-signaling pathways and regulates T-cell activation by bridging TCRs [7,9]. In our previous studies, we found Ser269/Thr291-phosphosites of *HIP-55* mediated the interaction with 14-3-3τ (data not shown), which was confirmed by a recent research of 14-3-3-phosphoproteome [24]. Further, we also found pro-oncogenic function of *HIP-55* through Ser269/Thr291-phospho-sensor motifs (data not shown).

## 3. Experimental Section

### 3.1. Chemicals and Materials

Sequencing-grade TPCK-modified trypsin was obtained from Promega (Madison, WI, USA). ACN, formic acid, TCEP, Supel-Tips Ti Pipette Tips were purchased from Sigma-Aldrich (St. Louis, MO, USA). EDTA-free protease inhibitor cocktail tablets were from Roche (Basel, Switzerland). Phosphatase inhibitor cocktail and Ni-NTA resin were from Pierce Biotechnology (Rockford, IL, USA). Anti-*HIP-55* antibody was purchased from BD Bioscience (San Diego, CA, USA). All other chemicals, as well as the Bradford protein assay kit, were from Bio-Rad (Hercules, CA, USA); the *HIP-55* plasmid was kindly provided by Prof. Haian Fu from Emory University. Ultra-pure water was prepared by a MilliQ water purification system (Millpore, Bedford, MA, USA).

### 3.2. Expression and Purification of HIP-55 Protein

The pDEST-His-*HIP-55* plasmid with full length human *HIP-55* gene was transfected transiently into HEK293 cells using Fugene HD (Roche) following the manufacturer’s protocol. Cells were lysed 36 h post-transfection in His pull-down lysis buffer (1% Nonidet P-40, 137 mM NaCl, 1 mM MgCl_2_, 40 mM Tris-Cl, 60 mM imidazole, 5 mM Na_4_P_2_O_7_, 5 mM NaF, 2 mM Na_3_VO_4_, 1 mM phenylmethylsulfonyl fluoride, 10 mg/L aprotinin, 10 mg/L leupeptin). The lysate was cleared by centrifugation and a small aliquot of the supernatant was removed for Western Blot analysis. Then the supernatant was incubated with Ni-NTA resin at 4 °C for 2 h, which was pre-equilibrated with a buffer containing 100 mmol/L PBS, 6 mol/L guanidine HCl and 10 mmol/L imidazole; pH 7.4. After washes by the same buffer, the proteins bound to the Ni-NTA resin were eluted with a buffer containing 100 mmol/L PBS, 6 mol/L guanidine HCl and 250 mmol/L imidazole; pH 7.4.

### 3.3. Tryptic Digestion

The eluted proteins were precipitated by adding four volumes of −40 °C acetone and kept at −20 °C for at least 2 h. Then the protein pellet was redissolved with a buffer containing 0.1% SDS and 50 mmol/L ammonium bicarbonate, pH 8.5. The protein sample was reduced at 60 °C for 30 min by adding TCEP to a final concentration of 5 mmol/L, followed by alkylation with iodoacetamide at a final concentration of 4 mmol/L in the dark at room temperature for 60 min. Then the protein samples were cooled down to room temperature and mixed with freshly prepared sequencing-grade TPCK-modified trypsin buffer (15 μg/mL in 25 mmol/L ammonium bicarbonate, pH 8.5) at an enzyme/protein ratio of 1:100. Digestion was performed at 37 °C for at least 15 h and stopped by adding 10% formic acid to a final concentration of 1%. The tryptic digests were cleared by centrifugation and the supernatant was purified over C18 tips. For enrichment of phosphopeptides, the supernatant that had not been processed by C18 tips was loaded onto TiO_2_ tips that were fully equilibrated with 3% formic acid. After washed with pure water three times, the bond peptides were eluted with 0.5% piperidine and then lyophilized.

### 3.4. Mass Spectrometry

The tryptic peptide samples were dissolved in buffer A (0.1% formic acid, 1% ACN), which were separated on a C18 reverse capillary column (150 mm × 0.17 mm) with buffer B (0.1% formic acid, 99% ACN) over a 60 min gradient of 2%–40%. The eluted peptides were then delivered into an LTQ Orbitrap mass spectrometer (Thermo Fisher Scientific, Waltham, MA, USA) equipped with a nanospray source. The mass spectrometer was operated in a data dependent mode, in which five MS/MS scans on the most abundant ions detected in the MS scan (400–1500 *m*/*z*) were acquired. The dynamic exclusion time was set as 1 min. Singly-charged ions were excluded for MS/MS.

### 3.5. Peptide and Protein Identification

The collected spectra were analyzed by Thermo Proteome Discoverer 1.1 software (Thermo Fisher Scientific, Waltham, MA, USA), in which the built-in SEQUEST as used to search the data against IPI protein database (International Protein Index, ipi.HUMAN.v3.84, downloaded at ftp://ftp.ebi.ac.uk/pub/databases/IPI) with the following parameters: trypsin was selected with two maximum missed cleavage allowed; precursor mass tolerance was 15 ppm; fragment mass tolerance was 0.8 Da; carbamidomethylation of cysteine was set as static modification; phosphorylations of serine, threonine and tyrosine, as well as oxidation of methionine, were set as dynamic modifications. The results from SEQUEST searches were filtered with Peptide Confidence as High, in which XCorr threshold was set as 1.9, 2.3 for doubly, triply-charged ion peaks, respectively.

### 3.6. Motif Analysis of Phosphosites

Sequence features adjacent to the identified phosphosites (serine, threonine, and tyrosine) were created and displayed using the Weblogo server (http://weblogo.berkeley.edu/).

### 3.7. SDS-PAGE and Immunoblotting

Cell lysates (25 μg) were subjected to electrophoresis in 12% Tris-glycine-SDS polyacrylamide gel using a Mini-Cell system (Bio-Rad, Hercules, CA, USA). Gels were electroblotted to 0.2 μm pore size polyvinylidene fluoride (PVDF) membranes (Millipore, Bedford, MA, USA). Then the membranes were blocked with 1% nonfat dry milk in a buffer containing 25 mM Tris, pH 7.5, 150 mM NaCl, 0.05% Tween 20 for 1 h at room temperature. Membranes were then incubated with the primary antibody against *HIP-55* (BD Bioscience, San Diego, CA, USA) for 2 h at room temperature. After washing for 10 min in TBST solution, membranes were incubated with properly diluted secondary antibody conjugated with horseradish peroxidase (Sigma-Aldrich, St. Louis, MO, USA) for 1 h at room temperature. Western signals were developed with ECL chemiluminescent reagents (GE, Boston, MA, USA).

## 4. Conclusions

In the present study, HEK293 cells over-expressing human *HIP-55* protein were used to establish a profile of phosphorylation sites within *HIP-55*. Ni-NTA resin enrichment and gel electrophoresis were utilized to collect the target protein and TiO_2_ affinity chromatography and biological mass spectrometry were employed to investigate phosphorylation. Investigation of the mass spectra revealed 14 phosphorylation sites of *HIP-55* protein, several of which were identified for the first time. Our findings provide evidence for future investigations of the phosphorylation status of *HIP-55* protein of different subtypes and facilitate further studies on their biological functions in health and disease.

## Figures and Tables

**Table 1.** Identification of phosphopeptides within *HIP-55* protein over-expressed in HEK293 cells.

Phosphopeptides	Experimental *m*/*z* (mono)	Theoretical *m*/*z* (mono)	Charge	XCorr	Position in protein
GACAS *HVSTMASFLK	795.353	795.346	2	1.98	95–109
GACAS *HVSTM *ASFLK	803.351	803.344	2	1.94	95–109
GAHVT *INAR	509.744	509.745	2	2.37	110–118
ASGANY *SFHKES *GR	835.828	835.822	2	2.21	135–148
TNAVS *EIK	471.224	471.221	2	3.06	165–172
VGKDS *FWAK	559.253	559.258	2	2.57	174–182
REQRY *QEQGGEAS *PQR	1039.924	1039.926	2	1.94	220–235
AMS *TTSISSPQPGK	736.333	736.329	2	3.63	267–280
AMS *TT *SISSPQPGK	776.318	776.312	2	2.56	267–280
AM *S *TTSISSPQPGK	744.324	744.326	2	3.23	267–280
LRS *PFLQK	534.785	534.784	2	2.41	281–288
QLT *QPETHFGR	697.321	697.317	2	2.01	289–299
QLT *QPET *HFGR	737.304	737.300	2	1.98	289–299
EPAAAIS *RPR	574.289	574.285	2	2.49	300–309

NOTE: T *, S * and Y * refer to the phosphorylated form of threonine (Thr, T), serine (Ser, S) and tyrosine (Tyr, Y), respectively; M * refers to the oxidized methionine (Met, M).
